# Supplementary material for: Phonological processing skills influence text-reading fluency in Russian-speaking adolescents
Source: PLoS One. 2025 Dec 5;20(12):e0337614. doi: 10.1371/journal.pone.0337614 (PMC12680206; doi:10.1371/journal.pone.0337614)
Supplement: S1 Table — (PDF) [file pone.0337614.s005.pdf]

S5 Table. Descriptive statistics for cognitive and reading tests.

Variable	Mean	SD
**Word reading fluency (words per minute)**	103.93	17.28
**Pseudoword reading fluency (pseudowords per minute)**	61.80	13.27
**Text reading fluency (words per minute)**	144.80	24.41
**Text comprehension score (number of correct answers out of 8)**	6.37	1.19
**IQ score (raw score in Raven's matrices)**	46.90	6.47
**Memory score (composite score out of 4)**	1.21	1.15
**Reading Attitude Index (composite score out of 10)**	7.19	2.19
