# Supplementary material for: Phonological processing skills influence text-reading fluency in Russian-speaking adolescents
Source: PLoS One. 2025 Dec 5;20(12):e0337614. doi: 10.1371/journal.pone.0337614 (PMC12680206; doi:10.1371/journal.pone.0337614)
Supplement: Attachment — Submitted filename: Response to Reviewers.docx [file pone.0337614.s007.docx]

PONE-D-25-32670

Phonological processing skills influence text-reading fluency in Russian-speaking adolescents

PLOS ONE

Dear Dr. Eremicheva,

Thank you for submitting your manuscript to PLOS ONE. After careful consideration, we feel that it has merit but does not fully meet PLOS ONE’s publication criteria as it currently stands. Therefore, we invite you to submit a revised version of the manuscript that addresses the points raised during the review process.

==============================

The manuscript presents valuable data on phonological processing and adolescent reading but is considered to require substantial revision to meet PLOS ONE’s publication standards. Both reviews converge on the need for clearer theoretical framing, stronger methodological transparency, and more rigorous statistical reporting. Key concerns include the ceiling effect in the CSP test, the limited validity of the proposed thresholds, insufficient description of materials and procedures, and underdeveloped analyses that do not fully support the claims.

A recommendation of **major revision** is being made. The authors are expected to clarify the theoretical model, reframe normative claims as exploratory, expand methodological detail (including task order, scoring, and inter-rater reliability), and strengthen statistical reporting (β coefficients, confidence intervals, model diagnostics). Claims of clinical or practical significance should be tempered in light of the small effect sizes observed. If these revisions are comprehensively addressed, the manuscript may be suitable for reconsideration.

==============================

Please submit your revised manuscript by Oct 31 2025 11:59PM. If you will need more time than this to complete your revisions, please reply to this message or contact the journal office at plosone@plos.org. When you're ready to submit your revision, log on to <https://www.editorialmanager.com/pone/> and select the 'Submissions Needing Revision' folder to locate your manuscript file.

Please include the following items when submitting your revised manuscript:

- A rebuttal letter that responds to each point raised by the academic editor and reviewer(s). You should upload this letter as a separate file labeled 'Response to Reviewers'.
- A marked-up copy of your manuscript that highlights changes made to the original version. You should upload this as a separate file labeled 'Revised Manuscript with Track Changes'.
- An unmarked version of your revised paper without tracked changes. You should upload this as a separate file labeled 'Manuscript'.

If you would like to make changes to your financial disclosure, please include your updated statement in your cover letter. Guidelines for resubmitting your figure files are available below the reviewer comments at the end of this letter.

If applicable, we recommend that you deposit your laboratory protocols in protocols.io to enhance the reproducibility of your results. Protocols.io assigns your protocol its own identifier (DOI) so that it can be cited independently in the future. For instructions see: [https://journals.plos.org/plosone/s/submission-guidelines#loc-laboratory-protocols](https://track.editorialmanager.com/CL0/https:%2F%2Fjournals.plos.org%2Fplosone%2Fs%2Fsubmission-guidelines%23loc-laboratory-protocols/1/010f019950d46add-9a51f52c-9775-470a-b87a-845655e643eb-000000/aaizW_ibJxxLkHIVUwjq3Lf8a0VQ8nif1aIn_DJy3uE=228). Additionally, PLOS ONE offers an option for publishing peer-reviewed Lab Protocol articles, which describe protocols hosted on protocols.io. Read more information on sharing protocols at [https://plos.org/protocols?utm_medium=editorial-email&utm_source=authorletters&utm_campaign=protocols](https://track.editorialmanager.com/CL0/https:%2F%2Fplos.org%2Fprotocols%3Futm_medium=editorial-email%26utm_source=authorletters%26utm_campaign=protocols/1/010f019950d46add-9a51f52c-9775-470a-b87a-845655e643eb-000000/37sRF3hlLqQ9M5rgMuT8V72m89OnDrn1MeGArUYTMG4=228).

We look forward to receiving your revised manuscript.

Kind regards,

Ramandeep Kaur
Academic Editor
PLOS ONE

Journal Requirements:

When submitting your revision, we need you to address these additional requirements.

1. Please ensure that your manuscript meets PLOS ONE's style requirements, including those for file naming. The PLOS ONE style templates can be found at

<https://journals.plos.org/plosone/s/file?id=wjVg/PLOSOne_formatting_sample_main_body.pdf>and

<https://journals.plos.org/plosone/s/file?id=ba62/PLOSOne_formatting_sample_title_authors_affiliations.pdf>

**Answer**: We ensured the journal’s requirements and revised our file naming.

2. Thank you for stating the following financial disclosure:
This research was supported by the Basic Research Program at the National Research University Higher School of Economics (HSE University).

**Please state what role the funders took in the study.** If the funders had no role, please state: "The funders had no role in study design, data collection and analysis, decision to publish, or preparation of the manuscript."
If this statement is not correct you must amend it as needed.
Please include this amended Role of Funder statement in your cover letter; we will change the online submission form on your behalf.

**Answer**: The funders had no role in study design, data collection and analysis, decision to publish, or preparation of the manuscript.

3. When completing the data availability statement of the submission form, you indicated that you will make your data available on acceptance. We strongly recommend all authors decide on a data sharing plan before acceptance, as the process can be lengthy and hold up publication timelines. Please note that, though access restrictions are acceptable now, your entire data will need to be made freely accessible if your manuscript is accepted for publication. This policy applies to all data except where public deposition would breach compliance with the protocol approved by your research ethics board. If you are unable to adhere to our open data policy, please kindly revise your statement to explain your reasoning and we will seek the editor's input on an exemption. Please be assured that, once you have provided your new statement, the assessment of your exemption will not hold up the peer review process.

**Answer**: Thank you for your concern! We updated the information on data acceptance. Our data is now freely accessible via the OSF platform: <https://osf.io/p6f24/overview?view_only=de243c43b160419783d68a5f171db6f5>

4. Please amend the manuscript submission data (via Edit Submission) to include author Yana Nikonova

**Answer**: Thank you for your comment! We included author Yana Nikonova in the submission data.

5. Please amend your authorship list in your manuscript file to include author Yana Nikonona

**Answer**: Thank you for your comment! We included author Yana Nikonova in the manuscript file.

6. If the reviewer comments include a recommendation to cite specific previously published works, please review and evaluate these publications to determine whether they are relevant and should be cited. There is no requirement to cite these works unless the editor has indicated otherwise.

**Answer**: Thank you for your comment! We did not receive any recommendations on the specific works that we need to cite in our manuscript.

Additional Editor Comments:

Thank you for submitting your manuscript on phonological processing skills and reading fluency in Russian-speaking adolescents. This is a valuable topic, and your dataset has the potential to make a meaningful contribution. However, substantial revisions are necessary before the manuscript can be considered for publication.

**Answer to the editor:** We sincerely thank you for your valuable comments and the opportunity to revise our manuscript. We carefully considered all the feedback provided and made substantial revisions to improve the clarity, structure, and overall quality of the paper.

A. Revisions Required for Consideration

**Theoretical Framework**

Clearly distinguish phonological processing components (e.g., PA, PWM) from the tests used to measure them (e.g., RAN, CSP).
Correctly situate RAN as a task rather than a component.

**Answer**: Thank you for the valuable comments on the features of the traditional PP model. We corrected the usage of the relevant terminology throughout the manuscript and accurately proposed the model’s components in the Introduction section (see page 4).

Explain how CSP maps onto specific PP components and how it differs from RAN in terms of cognitive processes.

**Answer**: We explained the features of the CSP test and its relation to the traditional PP model in the Introduction section (the paragraph devoted to This Study; see page 11).

Discuss the role of Russian orthographic and morphological features in interpreting the findings.

**Answer**: Thank you for the relevant suggestions on the role of Russian grammar in interpreting the results. The more detailed description can be seen in the Introduction section (the paragraph devoted to This Study; see pages 13-14).

**CSP Thresholds**

Reframe the reported thresholds as sample-referenced and exploratory, not normative, given the ceiling effects and small per-grade subsamples.

**Answer**: Thank you for the suggestions on the CSP thresholds. We corrected the reported information according to your comments. See the Results section (page 27-28) and the Discussion section (pages 32-33).

Justify the choice of a 10% cut-off and provide sensitivity analyses with alternative thresholds.

**Answer**: We thank the reviewer for this valuable comment. The rationale for selecting the 10% cut-off is now explained in the revised manuscript (page 27, paragraph Thresholds for phonological test).

**Methods Transparency**

Participants: Describe recruitment procedures and provide justification for inclusion/exclusion criteria.

**Answer**: We thank the reviewer for this comment. The recruitment procedures and inclusion/exclusion criteria are now described in detail in the revised manuscript (pages 14-16, paragraph Participants).

Clarify how gender was analyzed.

**Answer**: We appreciate the reviewer’s suggestion. The approach to analyzing gender is now clarified in the revised manuscript (pages 24-26, paragraph Data analysis).

Materials: Address similarity between word and pseudoword lists, and potential carryover effects.

**Answer**: Thank you for this important comment. We have addressed the similarity of word and pseudoword lists and discussed potential carryover effects in the manuscript (pages 16-17, paragraph Word and pseudoword reading tests).

Provide information about the text-reading passage (genre, curriculum relation).

**Answer**: We appreciate the reviewer’s attention to detail. Information on the text-reading passage, including its genre and relation to the curriculum, has been added (pages 17-18, paragraph Text reading test).

Procedure: Clarify task order, whether it was counterbalanced, and address possible fatigue or order effects.

**Answer**: Thank you for this comment. Task order, counterbalancing, and potential fatigue and order effects are now clarified in the revised manuscript (pages 21-24, section Procedure; pages 41-42, paragraph Limitations of our study and directions for further research).

Scoring: Provide information on number of raters, their training, inter-rater reliability, and rules for handling errors and self-corrections.

**Answer:** Thank you for the relevant comments on the scoring procedure! We decided to add a separate paragraph dedicated to the details of scoring in this study (see pages 23-24, paragraph Manual scoring)

**Statistical Analyses and Reporting**

Report β coefficients, standard errors, 95% confidence intervals, exact p-values, and adjusted R² values.

**Answer:** We thank the reviewer for this suggestion. We have now included a table reporting β coefficients, standard errors, 95% confidence intervals, exact p-values, and adjusted R² values for all three final models in the revised manuscript (Results section, Table 3).

Clarify your approach to multiple comparisons.

**Answer:** We appreciate the reviewer’s comment and acknowledge the importance of addressing multiple comparisons. In our study, we did not apply corrections for multiple comparisons, as we conducted three independent analyses. Word and pseudoword reading fluency cover two independent ways (sublexical and lexical, respectively) in the dual-route model (Coltheart et al., 2001); therefore, it is unlikely that they influence each other.

Regarding text reading fluency, although text reading engages mechanisms involved in both word and pseudoword reading (as readers may occasionally encounter unfamiliar words), we consider text reading fluency to be an independent variable. Text reading involves contextual and predictive processes that substantially change reading behavior compared to reading isolated words or pseudowords. Therefore, we treated these analyses as separate rather than multiple comparisons requiring correction.

Explore interactions (e.g., PP × grade, PP × gender) and test for non-linear effects, or justify their omission.

**Answer:** We thank the reviewer for this comment. Interactions (e.g., PP × grade, PP × gender) have been added to the models, and the results are now described in the Results section (pages 27-31). Regarding non-linear effects, we consider their inclusion unnecessary because most predictors do not have sufficient unique observations to support such analyses. The only predictor with a sufficiently large number of data is the IQ score, which is reasonably approximated as linear.

Provide descriptive statistics and distributions for all measures, including checks for ceiling/floor effects.

**Answer:** Thank you for this suggestion. Descriptive statistics and distributions for all measures are provided in the supplementary materials (S1-S4 Figs, S5 Table).

**Interpretation of Findings**

Temper conclusions to reflect the modest incremental variance explained (~2% for text fluency).

**Answer**: Thank you for the valuable comments on our manuscript! We considered your recommendations and provided more tempered conclusions on the effect size in the Discussion section of this manuscript (see pages 33-34, paragraph Phonological test and text-level reading; pages 40-41).

Discuss limitations posed by the CSP ceiling effect and the limited predictive power for word and pseudoword fluency.

**Answer:** Thank you for the valuable comments on our manuscript! We considered your recommendations and provided a more detailed discussion on the CSP ceiling effect in the Discussion section of this manuscript (see pages 38-40, paragraph Limitations of our study and directions for further research). The predictive power of word and pseudoword reading tests is discussed on pages 34-37 (paragraph Phonological test and word-level reading).

Consider the potential role of comprehension in interpreting text fluency results.

**Answer:** We thank the reviewer for this suggestion. We added comprehension question scores as a predictor in the model predicting text reading fluency; however, this variable did not reach statistical significance, so it wasn’t included in the final model (see the Results section, pages 28-31).

**Presentation and Language**

Condense the introduction to better highlight the research gap in adolescents.

**Answer**: Thank you for the valuable comments on our manuscript! We considered your recommendations and provided major revisions to the Introduction section of this manuscript (see pages 3-14).

Introduce technical terms clearly upon first mention.

**Answer**: Thank you for the comment! We considered your recommendation and provided more detail on the technical terms used in our manuscript.

Correct phrasing such as “words fluency” to “word fluency” throughout.

**Answer**: Thank you for the recommendation! We edited this term throughout the text of our manuscript.

Standardize reference formatting and ensure completeness.

**Answer**: Thank you for the valuable comments on our manuscript! We checked the reference style in our manuscript.

**B. Recommended Improvements**

Consider including or at least discussing alternative PP measures (e.g., RAN, spoonerisms) to complement CSP.

**Answer**: Thank you for the valuable comment on the alternative PP measures. We discussed this topic in more detail in the Discussion section of this manuscript (pages 39-41, paragraph Limitations of our study and directions for further research).

Explore the possibility that PP may interact with broader language or cognitive skills (e.g., vocabulary, comprehension).

**Answer**: We thank the reviewer for this suggestion. Based on the available data, we examined interactions of PP with participant’s grade, gender, CSP test scores, Raven’s Standard Progressive Matrices scores, total memory scores, reading habits index, and comprehension question scores. The results of these analyses are now reported in the revised manuscript (see Data analysis and Results sections, pages 24-26, 28-31).

Enhance visualizations by including distribution plots and gender-specific markers.

**Answer:** Thank you for this comment. Results, including distributions by gender, are presented in Fig. 2.

Recast “clinical relevance” claims more cautiously, focusing on text-level reading under cognitive load.

**Answer**: Thank you for the commentary on the relationship between PP and reading tests. We reframed the part on the cognitive load in the Discussion section (see pages 42-43).

**Reviewer #1: Reviewer Comments:**
Manuscript Title: Phonological processing skills influence text-reading fluency in Russian-speaking adolescents
Decision: Major Revision

Dear Authors,

Thank you for submitting your manuscript titled "Phonological processing skills influence text-reading fluency in Russian-speaking adolescents." Your study addresses an important and underexplored topic by investigating phonological processing (PP) skills and reading fluency in Russian-speaking adolescents. The novelty of examining PP effects simultaneously on words, pseudowords, and text reading, and providing normative thresholds for PP assessment in this population, represents a valuable contribution.

However, several major issues must be addressed before the manuscript can be considered for publication:

**General response:** We are sincerely grateful for your exceptionally detailed and thoughtful review. We truly appreciate your careful attention to our work and the valuable feedback. We have carefully considered and addressed all your comments and suggestions to the best of our ability.

1. Ceiling Effect in the PP Test:

* Approximately 75% of participants scored at the top of the CSP test, indicating that this PP test may not be sufficiently challenging for adolescents. Discuss the implications of this limitation and consider additional analyses or alternative tools.

**Answer**: Thank you for the valuable comments on our manuscript! We considered your recommendations and discussed the ceiling effect of the CSP test in the Discussion section of this manuscript (see pages 38-40, paragraph Limitations of our study and directions for further research).

2. Effect Size and Practical Significance:

* The manuscript reports that PP explained only \~2% of variance in text reading fluency and did not significantly predict word or pseudoword reading. This small effect size should be acknowledged, and the practical or clinical implications discussed. Consider addressing language-specific features of Russian that might influence these results.

**Answer**: Thank you for the valuable comments on our manuscript! We considered your recommendations and provided more tempered conclusions on the effect size in the Discussion section of this manuscript (see pages 33-34, paragraph Phonological test and text-level reading; pages 40-41). We also discussed language-specific features of Russian in more detail (see page 40-41).

3. Statistical Analyses:

* The regression models are relatively simple, and Adjusted R² values are low. The manuscript should report additional statistical details (β coefficients, standard errors, confidence intervals) and clarify whether corrections for multiple comparisons were applied.
* Consider exploring interactions, non-linear effects, or multilevel models to better capture potential influences of PP on reading outcomes.

**Answer:** We are grateful for these helpful suggestions. Additional statistical details, including β coefficients, standard errors, and 95% confidence intervals, are now reported for all predictors in the table summarizing the three final regression models (Results section, Table 3, pages 28-31). We considered potential non-linear effects of age and grade; however, most predictors do not have enough unique observations to justify non-linear modeling. No corrections for multiple comparisons were applied, as explained in the Data analysis section.

4. Discussion and Interpretation:

* Expand discussion of gender differences observed in word reading but not in other reading measures.

**Answer:** Thank you for this comment. We have expanded the discussion of gender differences observed in word reading, while noting the absence of such differences in pseudoword and text reading, in the Phonological test and word-level reading subsection of the Discussion (pages 36-37).

* Clarify the implications of the small PP effect on text reading and discuss limitations of using CSP as the sole PP measure.

**Answer**: Thank you for the valuable comments on our manuscript! We considered your recommendations and provided major revisions in the Discussion section of this manuscript (see pages 31-42).

* Provide concrete recommendations for future research, including adaptation of other PP assessments (CTOPP, RAN, Spoonerism tasks) for Russian adolescents.

**Answer:** Thank you for the valuable comments on our manuscript! We considered your recommendations and provided more detailed practical implications in the Discussion section of this manuscript (see pages 39-40).

Summary:

*The manuscript titled "Phonological processing skills influence text-reading fluency in Russian-speaking adolescents" presents valuable data and fills an important research gap, but substantial revisions are required to address methodological limitations, clarify findings, and strengthen statistical reporting.

**Answer**: Thank you for your recommendations! We carefully reviewed them and provided the necessary information on methodological limitations, more clarified findings and statistical reporting in the manuscript.


Introduction:
1. Length and Excessive Detail
The introduction is very lengthy and highly detailed. While comprehensiveness is appreciated, in a high-impact journal, introductions are typically concise (3–5 paragraphs) and should lead the reader quickly to the research gap. Sections such as “Phonological processing skills and reading in children” and “Phonological processing skills and reading in adolescents” are overly detailed with study-specific results, which could be summarized more effectively.

**Answer**: Thank you for the valuable comments on our manuscript! We considered your recommendations and provided major revisions to the Introduction section of this manuscript (see pages 3-14).

2. Structure and Logical Flow
The current structure sometimes reads as a list of prior studies rather than a coherent narrative. For example, after discussing PP in children, the manuscript delves into very specific study details and then moves to adolescents, which disrupts the logical flow. I recommend presenting a general framework for PP and its relevance to reading first, then clearly identifying the research gap in adolescents, broader skill, and finally stating the study aims, Russian.

**Answer**: Thank you for the valuable comments on our manuscript! We considered your recommendations and provided major revisions to the Introduction section of this manuscript (see pages 3-14).

3. Use of Technical Terms
Specialized terms such as “Dual Route Cascaded model”, PA, PWM, and RAN are introduced without sufficient explanation. While these may be familiar to experts, a brief clarifying sentence for each term upon first introduction would improve accessibility for a broader readership.

**Answer**: Thank you for the comment! We considered your recommendation and provided more detail on the technical terms used in our manuscript (see the Introduction section, pages 4-6).

4. Lack of Critical Perspective
The introduction mostly summarizes prior studies descriptively but does not critically evaluate their limitations. High-tier journals expect authors to actively highlight gaps in existing literature and justify the significance of the present study.

**Answer**: Thank you for the valuable comments on our manuscript! We considered your recommendations and provided major revisions to the Introduction section of this manuscript (see pages 3-14).

5. Presentation of Aims and Hypotheses
The study aims and hypotheses are presented only at the end of the introduction, after extensive details. These could be stated earlier and more clearly to orient the reader to the purpose of the research from the beginning.

**Answer**: Thank you for the valuable comments on our manuscript! We considered your recommendations and provided major revisions to the Introduction section of this manuscript (see pages 3-14).

6. References and Citations
Some references appear incomplete or inconsistently formatted (e.g., \[36]). The citation list and in-text references should be carefully checked for accuracy and consistency.

**Answer**: Thank you for the valuable comments on our manuscript! We checked the reference style in our manuscript.

7. Language and Style
Sentences are often long and complex, sometimes containing multiple ideas. This affects readability. Shorter, clearer sentences with one main idea each would improve clarity and reader engagement.

**Answer**: Thank you for the valuable comments on our manuscript! We considered your recommendations and provided major revisions to the Introduction section of this manuscript (see pages 3-14).

8. Limited discussion of language-specific characteristics
While the study focuses on Russian-speaking adolescents, the introduction does not adequately address structural and phonological features of Russian that may influence phonological processing (PP) and reading. Differences in orthographic transparency, phoneme-grapheme correspondence, and reading norms should be highlighted to justify the relevance of studying this population.

**Answer**: Thank you for the valuable comments on our manuscript! We considered your recommendations and provided more detailed information on language-specific features of Russian in the last paragraph of the Introduction section (see pages 13-14).

9. Insufficient integration of broader language and cognitive skills
The introduction frames PP primarily as a predictor of reading, but reading in adolescents involves interaction with other language and cognitive skills (e.g., grammar, vocabulary, text comprehension). A more comprehensive framework linking PP to these broader skills would strengthen the rationale for the study.

**Answer**: Thank you for the valuable comments on our manuscript! We considered your recommendations and provided more information on the role of other cognitive skills in reading (see the Introduction section; pages 10, 13).

10. Limited discussion of cognitive complexity in adolescent reading
While detailed for children, the introduction provides less explanation of the additional cognitive demands of reading in adolescents, such as processing longer and more complex texts. This is critical for understanding why PP alone may not fully predict reading outcomes in this age group.

**Answer**: Thank you for the valuable comments on our manuscript! We considered your recommendations and discussed cognitive requirements for reading in adolescents in the Introduction section of our manuscript (see pages 9-14).

11. Insufficient justification of the assessment instruments
Although RuToPP, STARS, and SARS are mentioned, the introduction does not clearly specify which tasks target PA, PWM, or other components of PP, nor the validity of these instruments for Russian-speaking adolescents. Clear justification for the choice of instruments is needed.

**Answer**: Thank you for the valuable comments on our manuscript! We considered your recommendations and provided more detailed information on the tests in the Introduction section (see pages 11-13) and in the Method section, paragraph about the Materials (see pages 16-20).

12. Lack of a coherent theoretical framework for adolescents
The traditional three-component PP model (PA, PWM, RAN) is referenced, but the introduction does not critically evaluate its applicability to adolescents or discuss potential limitations or modifications needed for this age group.

**Answer**: Thank you for the valuable comments on our manuscript! We considered your recommendations and discussed possible alternatives for using the traditional PP model in the Introduction section (see pages 7-10, the idea on the number of involved processes).

13. Limited discussion of practical or clinical implications
The introduction emphasizes theoretical significance but gives limited attention to potential applications in identifying reading difficulties or informing intervention programs for adolescents. Explicitly linking the study to practical outcomes would increase its relevance.

**Answer**: Thank you for the valuable comments on our manuscript! We considered your recommendations and provided the information on the practical outcomes in the Introduction section (see pages 7, 10, 12) and in the Discussion section (see pages X).

**Summary Recommendation**
Overall, the introduction provides a thorough overview, but it would benefit from condensation, clearer structure, stronger critical framing of prior work, and more prominent presentation of study aims and hypotheses. These revisions will help engage readers and strengthen the rationale for your study.
The introduction would benefit from a stronger focus on language-specific considerations, integration of broader linguistic and cognitive skills, justification of the assessment tools, and explicit discussion of theoretical and practical implications for adolescent reading. Addressing these points would enhance both the scientific rigor and applied relevance of the study.

**Answer**: Thank you for the detailed commentary on the Introduction section of our manuscript. We noted your suggestions and rewrote the Introduction section with major revisions (see pages 3-14).


Methods:
1. Participants

* The sample size (N=163) is adequate, but the sampling procedure is not fully described. Were schools randomly selected, or was participation voluntary? This affects the generalizability of the findings.

**Answer**: Thank you for your question! We described the recruitment process in more detail on pages 14-15 (paragraph Participants).

* Gender distribution is reported, but the approach to analyzing potential gender differences in reading or phonological skills is not clearly described.

**Answer:** Thank you for this comment. Gender was included as a factor in all regression models, with females as the reference category (see Data Analysis section, page 24-26). Differences between male and female participants in reading and phonological skills were assessed via these models, and the results are reported in the Results section (page 29, Table 3).

* The inclusion criterion of nonverbal intelligence is reasonable, but the rationale for excluding participants solely based on below-norm scores should be more transparent.
**Answer**: We appreciate the reviewer’s comment. We have clarified the rationale for excluding participants with below-norm nonverbal intelligence scores in the Methods section (pages 14-15, paragraph Participants).

2. Materials – Words and Pseudowords Reading Test

* The STARS test is adequately described, but the selection of lists C and D requires further justification. Why were only these lists used instead of counterbalancing across participants? This could lead to order or list effects.

**Answer**: Thank you for the clarification on the balancing of the reading tests! We used two lists from the STARS test battery based on the recommendations of its authors (Dorofeeva et al., 2021). We described these recommendations in more detail to justify our choice of the tests (see pages 16-17). We did not balance the order of the tests, since they were included in a bigger test session with a variety of different instruments. The procedure was long and time-consuming, therefore, we decided to use a fixed test order (to avoid possible inconsistencies and to compare the results on each test in all participants). For a comprehensive description of the test order see pages 21-23.

* Stimulus complexity is described in terms of frequency and syllable count, but additional information about phonotactic legality and morphological complexity would strengthen the assessment of appropriateness for adolescents.

**Answer**: Thank you for your comment! We provided the information on phonotactic legality and morphological complexity of the STARS test in the Methods section (pages 16-17).

3. Text Reading Test

* Using a novel text (“The Caring Flower”) to avoid prior familiarity is reasonable, but the standardization is deferred to a future article. Brief information on reliability and validity should be included here to ensure interpretability.

**Answer:** Thank you for the comment! Due to time and financial constraints, as well as the challenges associated with organizing longitudinal studies, it was not possible to assess test–retest reliability in this study. However, we provided the inter-rater reliability measures for this test in the Methods section (see pages 23-24).

* Comprehension questions include open- and closed-ended formats, but scoring procedures for open-ended responses and inter-rater reliability are not reported.

**Answer**: Thank you for the fair comments on the features of the text reading test! We explained them in more detail on pages 17-18. Moreover, we added the description of the scoring system in this test in the corresponding paragraph (see pages 23-24).

4. Phonological Processing Test (CSP from RuToPP)

* The selection of CSP as the most complex test is justified, but confirmation that ceiling effects are not present in adolescents is needed. Was a pilot study conducted?

**Answer**: Thank you for the question! The pilot study was not conducted; the CSP test was chosen based on the recommendation of its creator, and after we found no possible alternatives in Russian.

* The connection between the five speech processes and theoretical PP components (PA, PWM, RAN) should be made explicit.

**Answer**: Thank you for the comment on the theoretical aspects of PP! We provided relevant information on the included linguistic processes in the Introduction section (see page 11).

* Only a single PP measure is used, which limits the ability to examine different facets of phonological processing independently.

**Answer**: Thank you for your fair comment on the rationality of the chosen PP test. We chose this test, since there were no published alternatives for Russian-speaking adolescents, which were able to assess their PP skills on a detailed level. The CSP test involves different linguistic skills (i.e., input processing, phonological working memory, phonological analysis, operations with phoneme sequences, output processing). Therefore, we suggested that this test was able to provide comprehensive information on the execution of different PP skills. However, in our future studies we would like to add to the procedure other (more convenient) PP tests, such as a spoonerism test or a forward/ backward naming of digits/ phonemes or RAN tests. These tests will allow us to assess traditional components of PP in more detail. 

5. Procedure

* Task order is clear, but potential fatigue or order effects are not addressed. CSP was administered last; prior tasks may influence performance.

**Answer**: Thank you for the fair comment on the order effect! We addressed this issue in the limitations section of our study (see pages 41-42).

* Scoring methods for reading (handling self-corrections and errors) should be described in detail.

**Answer**: Thank you for the relevant comments on the scoring procedure! We decided to add a separate paragraph dedicated to the details of scoring in this study (see pages 23-24)


6. Data Analysis

* Further clarification is needed regarding coding of gender and treatment of grade as a numeric variable.
* Non-linear relationships between age/grade and reading/PP may be relevant and should be considered.
* Reporting effect sizes and confidence intervals would enhance interpretability.

**Answer:** Thank you for this comment. Grade was treated as a numeric variable because it represents an ordinal scale with evenly spaced levels (8-11). Gender was treated as a factor, with females as the reference. We considered potential non-linear effects of age and grade; however, most predictors do not have enough unique observations to justify non-linear modeling. Effect sizes and 95% confidence intervals for all predictors are now reported in the table summarizing the three final regression models (Results section, Table 3)

7. Clarity and Reproducibility

* Overall methods are detailed, but information on text standardization, comprehension scoring, task order, and scorer reliability should be expanded for full reproducibility.

**Answer**: Thank you for the valuable comments on our manuscript! We considered your recommendations and provided major revisions in the Methods section of this manuscript (see pages 14-26).


Summary Recommendation
The methods are thorough and well-organized. Authors should:

1. Provide additional justification for instrument selection and task order,
2. Include brief reliability and validity information for the text reading task,
3. Address potential ceiling, fatigue, and order effects in adolescents,
4. Expand statistical analysis reporting, including effect sizes and potential non-linear relationships,
5. Clarify scoring procedures and inter-rater reliability for reading and phonological tasks.

**Answer:** Thank you for the detailed and constructive feedback. We have carefully considered all suggestions and have revised the manuscript accordingly, including additional justification for instrument selection and task order, addressing potential ceiling, fatigue, and order effects, expanding statistical reporting with effect sizes, and clarifying scoring procedures and inter-rater reliability.

Results:
1. Descriptive statistics and data distribution

* Presenting both percentile-based and mean ± SD thresholds may be somewhat confusing. It would be clearer to select one primary metric for analysis and report the other as supplementary information.

**Answer**: We thank the reviewer for this suggestion. We have revised the section to present percentile-based thresholds as the primary descriptive metric (pages 27-28).

2. Regression models and variance explained

* Regression models for words, pseudowords, and text are appropriate, but the Adjusted R² values are very low (0.041–0.099), indicating that PP explains only a small portion of reading variance. This should be explicitly discussed to avoid overinterpretation.

**Answer:** Thank you for this comment. The low Adjusted R² values and limited variance explained by phonological processing are now noted in the Discussion section (see pages 31-42).

* For models where PP did not significantly predict word or pseudoword reading, additional analyses (e.g., non-linear models, interaction terms, or multilevel models) could be considered to determine whether small effects might emerge under more complex modeling.

**Answer:** We appreciate this suggestion. We explored potential interactions and non-linear effects; however, most predictors did not have enough unique observations to justify more complex modeling. These considerations are now noted in the Methods and Results sections.

3. Effects of gender and grade

* A significant gender effect is observed for word reading (females faster), but not for pseudowords or text reading. This discrepancy should be discussed, considering possible explanations such as scoring methods, motivation, or linguistic factors.

**Answer:** Thank you for this comment. We have discussed the significant gender effect. Possible explanations are now addressed in the Phonological test and word-level reading subsection of the Discussion.

* Grade level does not have a significant effect, but potential non-linear developmental changes across grades 8–11 should be considered.

**Answer:** Thank you for this comment. Grade level did not show a significant effect on reading outcomes. As previously noted, non-linear models were not applied because most predictors did not have enough unique observations to justify such modeling

4. Interpretation of PP effect on text reading

* PP significantly predicts text reading, but the additional variance explained is only \~2%. This is a very small effect and should be interpreted cautiously.
* Discussion should consider the practical or clinical relevance of this small effect.

**Answer**: Thank you for the recommendations on the possible improvements of our manuscript! We addressed these issues in the Discussion section (see pages 33-34, paragraph Phonological test and text-level reading; pages 40-41).

5. Clarity and reporting of data

* References to figures and tables are clear, but more detailed statistics (e.g., β coefficients, SE) would improve interpretability.
* Clarification on multiple comparison corrections or p-value adjustments, if any, should be provided.

**Answer:** Thank you for these helpful suggestions. Detailed statistical information, including β coefficients, standard errors, and 95% confidence intervals, is now provided in Table 3 (Results section). As explained in the Data analysis section, no corrections for multiple comparisons were applied, as the three analyses were treated as independent.

6. Overall summary

* Results are systematically and clearly presented. However, the effect of PP on reading is limited and small, and some models do not reach significance. The discussion should explicitly address these limitations and the small effect sizes, as well as developmental considerations.

**Answer**: Thank you for the valuable commentary on the Results section of this study. We considered your recommendations and provided a more detailed discussion of the obtained results in the Discussion section of this manuscript (see pages 31-42).

Discussion:


2. PP thresholds and practical application

* Limitations and generalizability of these thresholds to other populations (e.g., different geographic or socio-economic groups) should be discussed.

**Answer**: We thank the reviewer for this suggestion. We have revised the section to present percentile-based thresholds as the primary descriptive metric (pages 32-33).

3. PP and text-level reading

* The small effect size and cross-linguistic differences warrant more emphasis, including the practical significance of 2% variance explained.

**Answer**: Thank you for the valuable comments on our manuscript! We considered your recommendations and provided more tempered conclusions on the effect size in the Discussion section of this manuscript (see pages 33-34, paragraph Phonological test and text-level reading; pages 40-41).

* Comparison with English-language studies is logical, but a more detailed analysis of Russian orthography features (phoneme transparency, number of phonemes, morphological complexity) would strengthen interpretation.

**Answer**: Thank you for the valuable comments on our manuscript! We considered your recommendations and discussed language-specific features of Russian in more detail (see pages 40-41).

4. PP and word-level reading

* Reporting the lack of PP effect on words and pseudowords is clear, but the discussion could include deeper analysis of potential interactions with other cognitive or linguistic skills beyond lexical access.

**Answer**: Thank you for the valuable comments on our manuscript! We considered your recommendations and provided a more detailed description of the word-level effects in the Discussion section of this manuscript (see pages 34-37). We also discussed the effects of the other cognitive skills in the Discussion section (see page 38).

5. Gender differences

* While supported by prior literature, limitations related to sample size, cultural factors, and orthographic transparency of Russian should be acknowledged.

**Answer**: Thank you for the comments! We discussed the limits of sample size, socio-economic statuses and orthographic transparency of Russian in the Discussion section of this manuscript (see pages 31-42).

6. Limitations and future directions

* Main limitations, including small effect sizes and limited PP measures, should be emphasized.

**Answer**: Thank you for the valuable comments on our manuscript! We considered your recommendations and provided more tempered conclusions on the effect size in the Discussion section of this manuscript (see pages 33-34, paragraph Phonological test and text-level reading; pages 40-41).

* Future research suggestions could include using complementary RAN measures, examining interactions of PP with vocabulary or comprehension skills, and longitudinal reading assessment.

**Answer**: Thank you for the valuable comments on our manuscript! We considered your recommendations and addressed the usage of complimentary instruments in the Discussion section of this manuscript (see pages 40-41).

Limitations and Conclusion:

1. Limitations of the PP tool

* The recommendation to adapt or develop other tools (CTOPP, RAN, Spoonerism) is appropriate, but potential limitations in standardizing these tools for Russian should be discussed further.

**Answer**: We considered your recommendations and provided more detailed practical implications in the Discussion section of this manuscript (see pages 40-41).


3. Implications for text-level reading

* Findings suggest that PP can still influence text reading in adolescents, but the effect is small and may reflect overall cognitive load rather than specific phonological skills.
* The suggestion to investigate cognitive load in future research is well-founded, though the small effect size and practical relevance should be emphasized.

**Answer**: We considered your recommendations and discussed these issues in the Discussion section of this manuscript (see pages 31-42).

4. Clarity and future directions

* Limitations and conclusions are clearly presented, but more concrete recommendations for future research (e.g., developing more challenging tests for older adolescents) would strengthen the discussion.

**Answer**: We considered your recommendations and provided more detailed practical implications in the Discussion section of this manuscript (see pages 31-42).

Summary:

* Main limitation: ceiling effect and test sensitivity.
* PP affects text-level reading, but the effect is limited and may reflect overall cognitive load.
* Findings are consistent with the literature, and future research suggestions regarding tool development and cognitive load assessment are appropriate.

**Answer**: Thank you for the valuable comments on the limitations of this study. We addressed these issues in the Discussion section of this manuscript (see pages 31-42).

**Reviewer #2:** Review of Phonological processing skills influence text-reading fluency in Russian-speaking adolescents

Summary: The manuscript describes a study in which phonological processing skills, word, pseudoword, and text reading fluency were studied in four age groups of adolescents. The results showed that a test of phonological processing skills significantly predicted text reading fluency.

General evaluation: The introduction of the study convinced me that the research fills an important gap in focusing on how adolescents' reading skills might still be determined by their phonological skills. However, I think the sample size, especially the group sizes, is not suitable for providing normative data on the used PP test. I also do not agree with the author’s interpretation of the results when they compare the three regression models. The result and the method section could be improved by providing more information. The text also contains a few language mistakes that need to be corrected. The study is not appropriate for publication without major revisions.

**General Response:** Sincerely, thank you for your thorough and detailed review. We greatly appreciate the time and effort invested in providing such constructive feedback and have carefully considered all comments and suggestions, revising the manuscript accordingly to address them.

Review in details:
Introduction:
1) The introduction strongly focuses on which PP tests were found to be associated with different reading tests which is indeed an important question, but a clearer explanation of the processing during reading and how the different tests tap into these processes is missing. For example, the traditional model of PP (page 4) should be explained in a way that a difference is made between the components and the tests used to measure it, it should be explained how the different components relate to reading. For example, the RAN does not seem to be a component of any model (page 4), but a method to measure certain components of reading. Please, also correct this on page 7: “The third component of the traditional PP model, i.e., RAN…”.

**Answer**: Thank you for the valuable commentary on the introduction of the PP model and its relationship with reading. We applied major revisions to the Introduction section of this manuscript and provided a more extensive description of the PP model (see pages 3-6).

2) On page 9, it is not clear whether the chosen PP test only involves PA and PWM components or it might also require other processes. Moreover, I find it a bit controversial that somehow the introduction argues that the use of RAN test in other studies is a bit problematic because the RAN is a complex task, hence, it is less clear which processes predict reading skills when an association is found between reading fluency/skills and RAN. Then, the authors choose a PP test for the present study which is also complex. Hence, I wonder what do we gain from choosing the CSP test and not the RAN? Is it possible to explain the differences between the two tests in terms of underlying cognitive and linguistic processing?

**Answer**: Thank you for the valuable commentary on the justification of the CSP test as an instrument for the PP assessment. We provided more details about this test in the Introduction section of the manuscript (see the paragraph about This Study; page 11).

Methods:
3) Please, indicate whether the text used in the Text reading test was expository or narrative and whether it was part of school curriculum.

**Answer**: Thank you for this comment. We have clarified that the text used in the Text Reading test was narrative in nature and was not part of the school curriculum (pages 17-18).

4) On page 12, it is stated that “The list D included pseudowords which were created by taking existing Russian words from list C and replacing 1-3 graphemes in them.” This suggest to me that the words in two lists, measuring word and pseudo word fluency were similar. Is it so? And if yes, could similarities between the words of the two lists influence the results of the list which was given to the students as second?

**Answer**: Thank you for the clarification questions on the STARS test battery! We provided a broader description of this test based on the materials published by its authors (Dorofeeva et al., 2021). It is worth mentioning that authors replaced one grapheme only in the short words (consisting of 3-4 graphemes each), whereas in the longer words they replaced two or three graphemes. Thus, the developed pseudowords were not similar to the existing words, though they maintained the phonotactic legality. It resulted in the slower pseudoword reading fluency (which was expected) and the familiarity with the stimuli was not reported. The order of the tests was fixed for all the participants, therefore, possible order effects might be evident in all the results. We discussed the features of the STARS test battery in the Methods section (see the paragraph on the Materials, pages 16-17). The discussion of the order effects was added to the paragraph on the Procedure (pages 21-23) and to the Limitations section (page 42).

And a related question: what was the order of the words and pseudoword tests? This is not clear from the Procedure section on page 14. Which one was presented first? Was the order changes across participants? All participants got the same list of words for the two tasks?

**Answer**: Thank you for the questions, which help to clear the details of our procedure. On page 21 we added a more precise description of the test order. We also cleared that the order of the tests was fixed in all participants (including the order of the word and pseudoword tests). The participants received the same list of words (list C from the STARS test battery) and the same list of pseudowords (list D from the same test battery). It was described in the corresponding part of the Materials section (i.e., devoted to the STARS test battery; see pages 16-17).

5) Test evaluations: If I understand well performance of all tests needed to be evaluated by the researchers to get the final scores. Please, provide information on how many persons were involved in the scoring of the results. Inter-rater reliability should be provided (at least) on some part of the data of each task.

**Answer**: Thank you for the relevant comments on the scoring procedure! We decided to add a separate paragraph dedicated to the details of scoring in this study (see pages 23-24). Unfortunately, due to the time and financial constraints, as well as the challenges of organizing the longitudinal studies, it was possible to measure the test-retest reliability only for the CSP test.

6) Data analysis:
On page 16, the authors write that “The dependent, within-subject variables were words, pseudowords and text reading fluency”. As three independent regression models were run on the three response variables, i.e. the variables were not included in one statistical model, these are not really within-subject variables, just three different response variables from the same sample. Reading “within-subject variable” was a bit confusing when I tried to understand the description of the data-analysis.

**Answer**: Thank you for the fair comment on the description of our analysis. We apologize for this mistake, which we corrected in the revised version of the manuscript (see pages 24-26).

Results
7) Threshold for phonological test:
In general, a group of data with sample size between 31 and 55 is too low and not representative for any normalization and thresholding, especially, if it is not done together with other measures (e.g., clinical assessment of reading and language difficulties) which could reliably show that low performing participants on certain test have reading, and language difficulties confirmed by other sources as well.

**Answer**: Thank you for the valuable and fair comment! We took it into consideration and refrained the reported thresholds as sample-referenced and exploratory (in accordance with the reviewers’ and editor’s suggestion).

It is also not clear why 10% was chosen as a threshold for risk.

**Answer**: Thank you for the valuable comment! We chose the 10%-cutoff for indicating the participants at risk based on the standards provided in (World Health Organization, 2018). We added this information in the manuscript as well (see page 27).

8) There was a significant gender difference in word reading fluency but the Figure 2 does not show data colored/indicated by gender. Gender information should be added to Figure 2 or it should presented in the separate figure.

**Answer:** Thank you for this comment. We have updated Figure 2 to include gender information, with points and regression lines distinguished by gender.

9)There is no information on the descriptive statistics of any of the fluency task, although it would be important to see whether it is possible that there is any ceiling effect or if there might be differences in the variation between these variables.

**Answer:** Thank you for this comment. Descriptive statistics for all fluency tasks, including checks for potential ceiling effects and variation across variables, are now provided in S5 Table.

10) I do not find information whether the results of the comprehension test have been used for assessing validity of the text fluency test, and whether there was any tradeoff between fluency and comprehension. This would be important in the interpretation of the association between CSP test results and text reading fluency.

**Answer**: Thank you for this comment. We tested a model with text reading fluency as the dependent variable and comprehension as the predictor, but no significant influence was found. This aligns with previous studies showing that reading fluency is not always predicted by comprehension (Brysbaert, 2019; Kim et al., 2021).

Discussion
10) In general, I am not convinced that the provided threshold for the CSP test (see above) could be used to identify the potential risk of phonological deficit in adolescents.

**Answer**: We considered your commentary on the justification of the provided thresholds and rewrote this paragraph according to the suggested formulation. See pages 32-33.

11) On page 24, the authors give some reasons for the lack of the effect of the PP skills on the word-level fluency. I would also add that the word-fluence tasks might be less sensitive, i.e. there might be less variation in the word-fluency measures compared to the text fluency to show any reliable effect (i.e. if the majority of the participants actually easily could do the task, there is no variation to predict). To rule out this explanation, please, also add the information of the distribution of these variables (see my point above).

**Answer**: Thank you for the fair comments regarding the word and pseudoword reading tasks. We considered your suggestions and edited this part of the manuscript in the Discussion section (see pages 34-37). The distribution of the results in these tests is reported in S5 Table.

Conclusion
12) On page 26, the authors write: “the complex structure of the PP test suggest that it is not specific phonological skills, but rather the overall level of cognitive load that may significantly influence adolescents’ reading fluency”.
It is not clear to me why the authors rule out that some phonological processing skills measured by the PP test could be associated with text reading fluency. Is it because this effect was not found on the word- and pseudo-word level? In this case, please, write that also in the conclusion. Although I am still not convinced about strong conclusions could be made about the lack of effect on the word- and pseudo-word level, because of the lack of information about the results of these tests (see my point above). However, in any case, it would be nice to read more discussion on what are the underlying processes in the used PP test (CSP), and how those could influence reading.

**Answer**: Thank you for the commentary on the relationship between PP and reading tests. We reframed the part on the cognitive load in the Conclusions section (see page 43). We also provided the distribution of the results in the reading tests in S5 Table. The more detailed description of the CSP test and its underlying linguistic processes is presented in the Introduction section (see page 11) and in the Methods section (see pages 19-20).

13) On page 26 (last sentence): “Therefore, further studies might be devoted to the assessment of cognitive load during PP and other cognitive tests in adolescents and its impact on reading texts.” It is not “the cognitive load during PP or the cognitive tests” which impact reading texts, but the cognitive load in general. Please, rephrase this sentence.

**Answer**: Thank you for the comment! We rephrased the chosen sentence in the Conclusions section (page 43).

Language in general
14) I am not a native speaker of English, therefore, I will not comment on language in detail but it appeared to me that it is “word fluency” and not “words fluency”. Please, correct it throughout the paper.

**Answer**: Thank you for the recommendation! We edited this term throughout the text of our manuscript.
